# Cost implications of HIV retesting for verification in Africa

**DOI:** 10.1371/journal.pone.0218936

**Published:** 2019-07-01

**Authors:** Arielle Lasry, Mireille B. Kalou, Paul R. Young, Jacqueline Rurangirwa, Bharat Parekh, Stephanie Behel

**Affiliations:** Division of Global HIV & TB, Center for Global Health, Centers for Disease Control and Prevention, Atlanta, Georgia, United States of America; Boston University School of Public Health, UNITED STATES

## Abstract

**Introduction:**

HIV misdiagnosis leads to severe individual and public health consequences. Retesting for verification of all HIV-positive cases prior to antiretroviral therapy initiation can reduce HIV misdiagnosis, yet this practice has not been not widely implemented.

**Methods:**

We evaluated and compared the cost of retesting for verification of HIV seropositivity (*retesting*) to the cost of antiretroviral treatment (ART) for misdiagnosed cases in the absence of retesting (*no retesting*), from the perspective of the health care system. We estimated the number of misdiagnosed cases based on a review of misdiagnosis rates, and the number of positives persons needing ART initiation by 2020. We presented the total and per person costs of *retesting* as compared to *no retesting*, over a ten-year horizon, across 50 countries in Africa grouped by income level. We conducted univariate sensitivity analysis on all model input parameters, and threshold analysis to evaluate the parameter values where the total costs of *retesting* and the costs *no retesting* are equivalent. Cost data were adjusted to 2017 United States Dollars.

**Results and discussion:**

The estimated number of misdiagnoses, in the absence of retesting was 156,117, 52,720 and 29,884 for lower-income countries (LICs), lower-middle income countries (LMICs), and upper middle-income countries (UMICs), respectively, totaling 240,463 for Africa. Under the *retesting* scenario, costs per person initially diagnosed were: $40, $21, and $42, for LICs, LMICs, and UMICs, respectively. When retesting for verification is implemented, the savings in unnecessary ART were $125, $43, and $75 per person initially diagnosed, for LICs, LMICs, and UMICs, respectively. Over the ten-year horizon, the total costs under the *retesting* scenario, over all country income levels, was $475 million, and was $1.192 billion under the *no retesting* scenario, representing total estimated savings of $717 million in HIV treatment costs averted.

**Conclusions:**

Results show that to reduce HIV misdiagnosis, countries in Africa should implement the WHO’s recommendation of retesting for verification prior to ART initiation, as part of a comprehensive quality assurance program for HIV testing services.

## Introduction

HIV misdiagnosis can lead to severe individual and public health consequences. A false-positive HIV diagnosis may lead to stigma and discrimination, strains on family relationships and reproductive choices, and unnecessary lifelong use of medication [1–4]. From a public health perspective, HIV misdiagnosis can undermine the public’s trust in HIV test results and in testing programs, and, can lead to inefficient spending associated with costly antiretroviral treatment (ART) and large settlements from lawsuits brought on by cases of misdiagnosis [5–8].

The use of recommended HIV rapid testing algorithms, comprised of at least two consecutive reactive assays in a high prevalence settings, or at least three consecutive reactive assays in a low prevalence settings, minimizes false-positive results [9]. However, a variety of factors, including but not limited to: user error, poor recordkeeping, inadequate management and supervision, use of incorrect testing algorithms, cross-reactivity and over-interpretation of weak reactive results, contribute to HIV misdiagnoses [10–12]. In global settings, studies have reported HIV misdiagnosis rates ranging from less than 1% to more than 10% [13–18].

The World Health Organization (WHO) recommends retesting for verification of all HIV-positive cases prior to ART initiation, in order to reduce the frequency of HIV misdiagnosis and prevent unnecessary initiation of ART [9, 19–21]. The recommendation calls for repeating the same nationally validated rapid testing algorithm on all HIV cases initially diagnosed, using a second blood sample, and a different tester. Retesting for verification is additional and subsequent to the two or three assays used as part of a rapid HIV testing algorithm leading to an initial HIV-positive diagnosis. Retesting for verification applies only to persons not on ART because HIV diagnostic tests validated for use on persons taking ART are not available. And, once persons are on ART, rapid tests may give false negative results due to waning of antibodies [22–24].

WHO’s 2015 “Treat All” guidelines call for the immediate initiation of ART for all HIV-diagnosed persons, irrespective of their CD4 count. The benefits of “Treat All” include improved health outcomes for persons living with HIV, a more efficient linkage to treatment process and population benefit of reduction in transmission [25]. ART initiation upon testing positive for HIV, does not allow a health care provider to observe the natural history of CD4 levels and question the diagnosis in the absence of a CD4 decline. Therefore, the adoption of “Treat All” and the associated surge in ART initiations creates an imminent window of opportunity and impetus for adopting retesting for verification as a manageable component to strengthening the quality of HIV care services.

While WHO first recommended retesting for verification of new diagnoses in 1997, the recommendation has been re-emphasized with the introduction and adoption of the “Treat All” guidelines [26]. Retesting for verification has not been widely adopted nor implemented [27]. This slow adoption may be related to: lack of knowledge about the retesting recommendation; providers’ reliance on other clinical assessments as required under previous ART guidelines that were indicative of HIV infection; lack of data about the magnitude of misdiagnosis; concerns of the additional costs and resources needed to implement retesting for verification or suspicions that the operational requirements of re-testing could be an impediment to rapid ART initiation.

An analysis among pregnant women suggests that retesting is less expensive than treatment of those with false HIV-positive status [28]. A study of misdiagnosis also among pregnant women suggests that laboratory-based confirmation of HIV among those with an undetectable pre-ART viral load, is cost-saving when compared to lifetime cost of ART program enrolment for those misdiagnosed [29]. Another study of HIV misdiagnosis modeled the effect of retesting on a low- and high-prevalence cohort of 10,000 persons and suggests that the cost of retesting is recouped quickly when compared to the cost of ART for those misdiagnosed over 30 years [30]. Our study estimates the costs of implementing retesting across countries in Africa, and, to our knowledge, is the first cost analysis to base data inputs on reviews of misdiagnosis rates and testing costs, and the gap in the number of persons needing to be initiated on ART to reach 90% coverage by 2020.

We conducted a cost analysis to estimate and compare the total costs of implementing the WHO HIV retesting for verification recommendation in Africa, to the costs of not retesting. We define the positive result of an initial testing algorithm, as an initial diagnosis, and the positive result following retesting for verification as a verified diagnosis. We do not use the terms confirmatory testing or confirmed diagnosis to avoid confusion. We compared the cost of retesting individuals for verification of an initial diagnosis, to the cost of ART associated with the cases who would be HIV misdiagnosed, without retesting for verification. With this model-based evaluation, we aim to provide policy-makers in governments of low- and middle-income countries, and other resource-constrained settings, with evidence to support their decision-making process around HIV retesting for verification strategies.

## Methods

We conducted a cost analysis of HIV retesting for verification, from the perspective of the health care provider. We compared two scenarios: *retesting*, implementing retesting for verification of all initially diagnosed HIV-positive cases prior to ART initiation; and *no retesting*, which is the current practice in most developing countries [27]. In terms of ART costs, we considered only the costs incurred for those misdiagnosed as HIV-positive because the cost of ART for true positives would be the same in both scenarios. We considered all countries in Africa listed as member states of the United Nations African Group, except Equatorial Guinea, and the Seychelles, Cape Verde and São Tomé & Príncipe islands [31]. We presented both the total costs and per person cost of these scenarios for these 50 African countries, grouped by income level: 26 low-income countries (LICs), 15 lower-middle income countries (LMICs) and 9 upper middle-income countries (UMICs) [31]. Regions outside of Africa were not evaluated due to paucity of data.

We assumed retesting for verification would largely be conducted in facility-based settings, even when the initial diagnosis is provided in community-based settings because the recommendations for retesting call for a different person to administer the verification test. We used facility-based testing costs, which do not typically include recruitment and mobilization efforts, to approximate retesting costs. We reviewed the literature for studies, set in African countries, published in the last ten years and reporting facility-based HIV testing costs per person. We searched PubMed using the terms: cost, HIV, testing, and facility, and limited the publication year from 2007 to present. To identify further literature, we searched reference lists. We identified five relevant studies [32–36], from two systematic review of HIV testing costs [10, 37], and an additional three relevant studies published subsequent to those systematic reviews [38–40]. We present the facility-based cost per person tested resulting from these eight studies in Table 1. HIV counseling and testing services in these studies are largely provided by trained counselors, occasionally supported by nurses or laboratory assistants [35, 36, 40], and all are using rapid diagnosis testing in the initial algorithm. All eight studies considered costs from the perspective of the heath care provider, and, all but one study [32] adopted an economic costing approach. For each of the three income level country groups, we used the average cost per person tested to estimate the cost of retesting for verification, in our base-case analysis. In sensitivity analysis, we varied the retesting costs, between the lowest and the highest reported facility-based cost per person tested, by country income level.

**Table 1:** Review of facility-based HIV testing costs per person in Africa (2017 USD).

Income level / Country	Facility-based cost per person tested	Source
Low-income countries in Africa		
Uganda	14	Menzies, 2009 [34]
Uganda	7	Mulogo, 2013 [35]
Malawi	9	Maheswaran, 2016 [39]
Rwanda	5	Bautista-Arredondo, 2016 [79]
Lower-middle income countries in Africa		
Kenya	6	Obure, 2012 [36]
Swazi	9	Obure, 2012 [36]
Kenya	7	Bautista-Arredondo, 2016 [79]
Zambia	20	Bautista-Arredondo, 2016 [79]
Upper-middle income countries in Africa		
South Africa	9	Bassett, 2007 [33]
Nigeria	8	Aliyu, 2012 [32]
South Africa	43	Tabana, 2015 [40]
South Africa	33	Bautista-Arredondo, 2016 [79]

To estimate the resources needed to fund the 2016 UNAIDS Fast-Track Approach, Stover et al. conducted a review of published ART cost studies [41, 42]. We used this review, limited to Africa, to estimate the average annual cost of ART for each of the three income level country groups. The average annual cost of ART per client included antiretroviral drugs, other drugs, laboratory services and other service delivery costs. All cost data were adjusted to 2017 United States Dollars [43]. In sensitivity analysis, we varied these costs between the lowest and the highest reported average annual cost of ART, by country income level.

A recent systematic review found 30 studies reporting a false positive HIV diagnostic error rate, with a median rate of 3.1% [12]. We used a subset of those studies to determine the false positive misdiagnosis rate following an initial testing algorithm. From those 30 studies, we excluded seven not set in Africa [44–50]; ten were excluded for reporting on misdiagnosis following discordant rapid test results [16, 51–58], including use of a tie-breaker algorithm which are known to have lower specificity [13]. Further reasons for exclusion were reporting on acute infection among negative samples [59], and reporting on oral fluid testing [60], or insufficient data [61]. The remaining 11 studies were deemed relevant and allowed for pooling of the individual study estimates [14, 15, 17, 29, 62–67]. For each country income level, we estimated a weighted mean false positive misdiagnosis rate by summing the number of false positives reported across studies and dividing by the number of positives, also summed across studies. These data are presented in Table 2. For LICs, the misdiagnosis rate ranged from 0.7% to 10% and the weighted mean was 2.7%. For LMICs, the misdiagnosis rate ranged from 0.3% to 5% and the weighted mean was 1.1%. For UMICs, the misdiagnosis rate ranged from 0.3% to 2% and the weighted mean was 0.85%. In sensitivity analysis, we evaluated the results across these ranges.

**Table 2:** Review of false positive misdiagnosis in Africa.

Income level / Country	Number of false positives	Number of positives	Rate of positive misdiagnosis	Source
Low-income countries (LIC) in Africa				
DRC	24	229	10.5%	Klarkowski, 2009 [15]
Mozambique	24	3,223	0.7%	Nelson, 2016 [17]
DRC	34	330	10.3%	Shanks, 2013 [14]
Ethiopia	37	802	4.6%	Shanks, 2013 [14]
Burundi	2	121	1.7%	Shanks, 2013 [14]
Ethiopia	17	423	4.0%	Shanks, 2015 [13]
LIC Total	138	5,128	2.7%	
Lower-middle income countries (LMIC) in Africa				
Nigeria	1	318	0.3%	Manak, 2015 [62]
Cameroon*	-	187	5.1%	Aghokeng, 2009 [67]
Zambia	19	1,484	1.3%	Bock, 2017 [63]
Swaziland	14	2,533	0.6%	Khan, 2017 [64]
LMIC Total	53	4,709	1.1%	
Upper-middle income countries (UMIC) in Africa				
South Africa	1	241	0.4%	Bock, 2017 [63]
South Africa	3	952	0.3%	Hsiao, 2017 [29]
South Africa	2	299	0.7%	Kufa, 2017 [65]
Botswana	11	515	2.1%	Mine, 2015 [66]
UMIC Total	18	2,008	0.85%	

* This study did not report the absolute number of false positives identified. Therefore, we extrapolated the number of false positives based on the sample size and the reported specificity of the two rapid testing algorithms presented in the study.

Few studies indicate the reduction in misdiagnoses that could be obtained from retesting. Reports from Malawi show that misdiagnosis has decreased from 7% to 1% following the implementation of retesting for verification and related quality assurance measures [68]. Another study reported that re-testing for verification prevented six out of eight false positive misdiagnoses [63]. In our model, we assumed a 75% reduction in misdiagnoses would result from retesting for verification, and varied this parameter in sensitivity analysis and threshold analysis. Varying the reduction in misdiagnosis following retesting can also serve to capture any loss to follow-up attributable to the extra step of retesting for verification, and to correct any overestimate resulting from the concurrent effects associated with the implementation of other quality improvement measures.

To close the HIV treatment gap and reach 90% ART coverage by 2020, UNAIDS reports the number of persons who would need to be initiated on ART, by 2020 and by country [42]. We used these data to estimate the number of initially diagnosed HIV-positive persons who are not on ART, and the total ART costs of the projected misdiagnosed cases, for each of the three country income level groups. Our analysis is presented in the context of “Treat All” and assumes that all those diagnosed as HIV-positive, would be initiated and incur ART costs.

We define costs per person initially diagnosed as the average per person costs across the total number of initially diagnosed, ART-naïve HIV cases, prior to retesting for verification. We estimate the per person costs and total costs of the *retesting* and *no retesting* scenarios over a ten year time horizon, as the base case. In sensitivity analysis, we explored a 5-year and 20-year time horizon. We assumed that all those initially-diagnosed HIV-positive persons who are not on ART would incur costs from the start of the time horizon. Future costs were discounted at rate of 3%.

To gauge the robustness of the findings, we conducted univariate sensitivity analysis and threshold analysis on the model input parameters. All input values for the base-case analysis and ranges examined in sensitivity analysis are presented in Table 3.

**Table 3:** Base-case values for model input parameters and range for sensitivity analysis.

	Low-income countries in Africa	Lower-middle income countries in Africa	Upper-middle income countries in Africa	Source
Average per person cost of HIV retesting for verification* & (*Range examined in sensitivity analysis)* (2017 USD)	9 *(5–14)*	11 *(6–20)*	23 *(8–43)*	[32–36, 39, 40, 75, 76, 79]
Average annual cost of ART†, per client & (*Range examined in sensitivity analysis)* (2017 USD)	532 *(149–1*,*397)*	432 *(205–1*,*115)*	1,016 *(257–1*,*970)*	[42]
Misdiagnosis rate at initial diagnosis & (*Range examined in sensitivity analysis)* (%)	2.7 *(0*.*7–10*.*5)*	1.1 *(0*.*3–5*.*1)*	0.85 *(0*.*3–2*.*1)*	[13–15, 17, 29, 62–67]
Reduction in misdiagnosis rate following *retesting* & (*Range examined in sensitivity analysis)* (%)	75 *(50–100)*	75 *(50–100)*	75 *(50–100)*	[63, 68]
Number of positives to be initiated on ART† assuming 90-90-90 (by 2020)	5,801,212	4,684,128	3,528,093	[42]

*Retesting for verification costs are based on facility-based HIV testing services.

†ART = Antiretroviral treatment

## Results and discussion

Using the base-case values, total costs under the *no retesting* scenario exceed the total costs under the *retesting* scenario in all three country groups, suggesting that cost savings are associated with the adoption of retesting for verification. Over the ten-year horizon, the estimated number of misdiagnoses in the absence of retesting for verification was 156,117, 52,720 and 29,884, for LICs, LMICs and UMICs, respectively, totaling 238,721 for Africa. Using a 75% reduction in misdiagnosis from retesting for verification, the estimated number of misdiagnosed cases with retesting for verification was 39,029, 13,180 and 7,471 for LICs, LMICs and UMICs, respectively, totaling 59,680 for Africa, over the ten-year horizon.

The total cost of treatment for those misdiagnosed cases under the *no retesting* scenario was $727 million, $199 million, and $266 million (2017 United States Dollars (USD)) for LICs, LMICs and UMICs, respectively, over the ten-year horizon. And, costs per person initially diagnosed under the *no retesting* scenario, defined as the total costs divided by the estimated of number of positives to be initiated on ART, were: $125, $43, and $75 for (2017 USD) for LICs, LMICs, and UMICs, respectively. Total costs under the *retesting* scenario include the cost of retesting all positives and the costs of ART for those who would be misdiagnosed in spite of the retesting efforts; these are: $231 million, $98 million, and $146 million (2017 USD) for LICs, LMICs and UMICs, respectively. Costs per person initially diagnosed under the *retesting* scenario, defined as the total costs divided by the estimated of number of positives retested, were: $40, $21, and $42 for (2017 USD) for LICs, LMICs, and UMICs, respectively. When comparing, the two scenarios, the estimated savings from *retesting* totals $717 million over the ten-year horizon, across all country income levels when compared to the *no retesting* scenario. And, the savings per person retested are $85, $21, and $33 for LICs, LMICs, and UMICs, respectively, when retesting for verification is implemented. Results of the base-case analysis are presented in Table 4.

**Table 4:** Costs under *retesting* and *no retesting* scenarios, over ten-year time horizon (2017 USD).

	Low-income countries in Africa	Lower-middle income countries in Africa	Upper-middle income countries in Africa	Total
***No retesting***				
Estimated number of misdiagnoses	156,117	52,720	29,884	238,721
Total cost*	726,795,142	199,347,111	265,828,995	1,191,971,248
Cost per person initially diagnosed†	125	43	75	85
***Retesting***				
Estimated number of misdiagnoses	39,029	13,180	7,471	59,680
Cost of retesting all positives^‡^	49,135,803	47,940,900	79,458,610	176,535,314
Cost of ART for misdiagnosed cases^§^	181,698,785	49,836,778	66,457,249	297,992,812
Total cost^‖^	230,834,589	97,777,678	145,915,859	474,528,125
Cost per person initially diagnosed†	40	21	42	34
**Incremental cost (savings) of *retesting***^¶^	(495,960,553)	(101,569,433)	(117,168,153)	(717,443,122)
Incremental cost (savings) per person initially diagnosed †	(85)	(21)	(33)	(51)

*Represents the ten-year cumulative discounted costs of ART for those who would be misdiagnosed among all those estimated to initiate ART between 2017 and 2020 (assuming 90-90-90). Only the cost of ART incurred for those misdiagnosed as HIV-positive are considered because the cost ART for true positives would be the same in both the *No retesting* and *retesting* scenarios.

† Represents the corresponding total cost (savings) divided by the estimated of number of positives to be initiated on ART between 2017 and 2020 assuming 90-90-90

^‡^ Represents the discounted cost of retesting all those estimated to initiate ART between 2017 and 2020 (assuming 90-90-90); retesting refers to the routine retesting for verification of all initially diagnosed HIV positive cases prior to ART initiation.

^§^ Represents the ten-year cumulative discounted costs of ART for those who would be misdiagnosed in spite of the retesting for verification effort.

^‖^ Total cost under *retesting* = Cost of retesting all positives + Cost ART for misdiagnosed cases

^¶^ Incremental cost (savings) of *retesting =* Total cost under *retesting*—Total cost under *no retesting*

In one-way sensitivity analysis, we varied the base-case input parameters, across the ranges indicated in Table 3, and the time horizon to 5 and 20 years, for all three country groups. In all sensitivity analysis scenarios explored, the total cost of *no retesting* exceeded the total cost of *retesting*, with three exceptions. First, at a per person cost of treatment as low as $257, the estimated cost of *retesting* in UMICs would exceed the cost *no retesting* by $29 million. Also, in both LMICs and UMICs, the lower bound on the rate of positive misdiagnosis causes the cost of *retesting* to exceed the cost *no retesting* by $6 million and $5 million, respectively.

We conducted threshold analysis on the input parameters to establish the parameter values for which for the total cost of both scenarios would be the same. For the total cost of *retesting* to equal the total cost of *no retesting*, with other parameters held constant, the per person cost of HIV retesting would have to increase from $9 to $98 in LICs, from $11 to $33 in LMICs, and from $23 to $58 in UMICs. Also, for the total costs to be the same in both scenarios, the mean annual cost of ART would have to decrease from $532 to $48 in LICs, from $432 to $139 in LMICs, and from $1,016 to $405 in UMICs. Moreover, for the total cost of *retesting* to equal the total cost of *no retesting*, with other parameters held constant, the misdiagnosis rate at initial diagnosis would have to be reduced to less than 0.2% in LICs, less than 0.4% in LMICs, and less than 0.3% in UMICs. Alternatively, the reduction in misdiagnosis rate following retesting would have to be reduced from 75% to less than 30%, 24% and 7%, in UMICs, LMICs, and LICs, respectively. Lastly, the cost of HIV treatment for misdiagnosed cases would have to be cumulated over a time horizon of less than ten months for the total cost of *retesting* to equal the total cost of *no retesting* in LICs, less than 3 years in LMICs, and, less than four years for UMICs. Results of the sensitivity analyses and threshold analyses are presented in Table 5.

**Table 5:** Sensitivity and threshold analyses of *retesting* by low-income countries (LIC), lower-middle income countries (LMIC), and upper-middle income countries (UMIC) in Africa.

Variable modified				Total cost (savings)* (2017 USD)		
	LIC	LMIC	UMIC	LIC	LMIC	UMIC
Average per person cost of testing (2015 USD)						
Base Case	9	11	23	(495,960,553)	(101,569,433)	(119,913,136)
Lower bound	5	6	8	(515,723,617)	(120,557,298)	(171,441,919)
Upper bound	14	20	43	(469,731,330)	(59,398,657)	(54,164,278)
Threshold	98	33	58	-	-	-
Average annual cost of ART, per client (2015 USD)						
Base Case	532	432	1,016	(495,960,553)	(101,569,433)	(119,913,136)
Lower bound	149	205	257	(103,561,332)	(23,004,561)	29,042,481
Upper bound	1,397	1,115	1,970	(1,382,527,946)	(338,091,169)	(306,905,420)
Threshold	48	139	405	-	-	-
Misdiagnosis rate at initial diagnosis						
Base Case	2.7%	1.1%	0.85%	(495,960,553)	(101,569,433)	(119,913,136)
Lower bound	0.7%	0.3%	0.3%	(101,696,077)	6,167,780	5,285,521
Upper bound	10.5%	5.1%	2.1%	(2,073,707,649)	(626,907,200)	(423,286,111)
Threshold	0.2%	0.4%	0.3%	-	-	-
Reduction in misdiagnosis rate following *retesting*						
Base Case	75%	75%	75%	(495,960,553)	(101,569,433)	(119,913,136)
Lower bound	50%	50%	50%	(314,261,768)	(51,732,656)	(53,455,887)
Upper bound	100%	100%	100%	(677,659,339)	(151,406,211)	(186,370,384)
Threshold	7%	24%	30%	-	-	-
Time Horizon (years)						
Base Case	10	10	10	(495,960,553)	(101,569,433)	(119,913,136)
Lower bound	5	5	5	(244,126,357)	(32,495,747)	(27,803,506)
Upper bound	20	20	20	(897,927,758)	(211,821,960)	(266,934,671)
Threshold	0.8	2.8	3.5	-	-	-

* Total cost (savings) of *retesting =* Total cost under *retesting*—Total cost under *no retesting*

Our results indicate that adopting retesting for verification leads to significant savings in medical costs averted, even under conservative cost and outcome assumptions. While there is uncertainty in the value of the model’s input parameters, sensitivity analyses and threshold analyses demonstrate that the conclusion holds under a majority of scenarios. For each scenario, reviewing where threshold lies relative to the range defined by the lower and upper bound in sensitivity analysis, provides an indication of the stability of the analysis. For LICs, all threshold values lie considerably outside of the range explored in sensitivity analysis indicating that reasonable variations in parameter input values, would not alter the findings; this is critical because of the three country groups considered, LICs have both the highest rate of positive misdiagnosis and the most number of positives to be initiated, and thus the greatest potential for misdiagnosis in the absence of retesting. Among LMICs, the threshold value for misdiagnosis rate is 0.36%, and a lower bound of 0.31% misdiagnosis was set based on a study, in Nigeria which found one misdiagnosis among 318 tested [62]. All other ranges of input values explored for LMICs excluded the threshold. Lastly, for UMICs, the threshold for misdiagnosis rate was 0.34% which lies faintly beyond the lower bound of 0.32%, and the threshold for the treatment costs was $405 while the lower bound in was $257. These trends in country income levels suggests that, while *retesting* is cost-saving, the case for retesting is greater where resources are more constrained. It is possible, but not known, whether any correlation exists between country income level and misdiagnosis rates. Better resourced countries may be able to afford more quality assurance activities such as training and proficiency testing.

Our findings are also consistent with those of a recent analysis by Eaton, in spite of the different approach taken [30]. Our analysis considers discounted ART costs over ten years, rather than a 30-year time horizon, by which antibody and viral load testing technologies are likely to have changed the landscape of HIV diagnosis and treatment. Our study also benefited from a systematic review to establish a baseline rate and range for false positive diagnosis [12], while the aforementioned study relied on assumptions based on rapid test specificity data. Lastly, our study provides the total costs of retesting using the estimated numbers of persons requiring ART initiation across 50 countries in Africa, grouped by income level, while the Eaton study evaluates the costs of retesting assuming a 1% and 10% HIV prevalence among a cohort of 10,000. In spite of these differences both analyses, along with another [28], suggest that, at present, retesting for verification is cost-saving relative to the treatment of false positive HIV cases.

Our analysis has limitations. First, the costs considered are those incurred by the health care provider only, and broader societal costs were not considered. At the individual level, the consequences of an HIV diagnosis may include stigma and discrimination, violence, psychological trauma, and productivity losses; and it can influence partner, reproductive, and professional choices [1–3, 8]. Published case reports and media accounts of HIV misdiagnosis suggest that significant jury awards have been made to victims of HIV misdiagnosis [3, 5–7] and this negative publicity can lead to credibility loss for the public health system and its providers. We did not attempt to quantify or cost the effects of these repercussions. However, considering these costs would increase the total cost of *no retesting*, and would serve to encourage the implementation of HIV retesting for verification prior to ART initiation. Second, the model considered the cost of the retesting events but did not consider the costs associated with the adoption and rollout of a retesting for verification strategy such training and dissemination. Training is an integral component of introducing new operating procedures in HIV testing, and, all HIV testing services must be implemented along with supervision and quality assurance programs. Third, retesting would call for a two-test algorithm, in high prevalence settings, or a three-test algorithm, in low prevalence settings. To save time and labor costs when retesting, we assumed that all tests would be run in parallel, and therefore the impact on cost of a two- vs. three-test algorithm is the cost of the rapid test kit itself, which is typically a small fraction of testing costs. Also, in our analysis, retesting for verification was defined as repeating the same testing algorithm. And, retesting can rule out possible technical or clerical errors, including specimen mix-up through mislabeling and transcription errors, as well as random error by either the provider or the test device, though retesting for verification will not exclude misdiagnosis related to poor choice of a testing algorithm or cross-reactivity[18]. However, this risk should be reduced assuming the testing algorithm used is validated[20, 69, 70]. The WHO recommends following the approved national HIV testing algorithm and ensuring adherence to a quality-testing program with continuous quality assurance.

Retesting may provide significant correction from multiple sources of errors including, lack of training, quality assurance and over interpretation of weakly reactively test lines, as a different provider should be reading the results using a new blood sample [12] [20]. Retesting is common for diseases, including HIV, by reference laboratories in Western countries, but was not practiced routinely in resource-limited countries. Given the wide adoption of “Treat All” guidelines, it is critical to incorporate retesting for verification as part of quality assurance programs, to avoid misdiagnosis and unnecessary costs. Other approaches to prevent misclassification include viral load testing, and supplemental HIV testing methods, that are more expensive and more specific than a rapid testing algorithm. We did not explicitly model these approaches as they are used to address different points in the diagnostic error cascade from those addressed by retesting. Viral load testing, may further confirm a positive diagnosis when provided to ART-naïve individuals, is not a substitute for HIV retesting for verification because it addressed a different portion of errors. Also, an undetectable viral load at baseline cannot be used to rule out infection because up to 10% of HIV-positive persons may have an undetectable viral load as their initial baseline measurement, without having been on ART [71–74]. Therefore, an undetectable viral load at baseline can include both true HIV-positive and false positives. And, the turnaround time to obtain viral load testing results is, at present, much greater than that of obtaining results from retesting using a rapid HIV testing algorithm, and any delay introduced in getting patients diagnosed and initiated on treatment increases the risk of losing patients to follow-up and puts the achievement of 90% ART coverage by 2020 in peril. Lastly, in low- to middle-income country settings, there is little capacity to provide viral load testing prior to initiation of ART, and it will take several years to ramp up this capacity sufficiently to cover all newly diagnosed HIV cases [75, 76]. In the future, viral load testing may have a role in diagnosing HIV, but at present, given current technology and capacity, detection of HIV antibody testing is the most appropriate method to diagnose and confirm infection, especially in developing countries.

## Conclusion

In sum, this analysis suggests significant savings in HIV treatment costs averted from the adoption of WHO’s retesting for verification guidelines and the consequent reduction in misdiagnosis. Our study ought to drive attention to the incorporation of retesting for verification as part of quality assurance for “Treat All” services, and to the policy and operational needs for routinizing retesting.

Beyond adoption of the guidelines, participating in continuous quality improvement and adhering to the nationally validated HIV testing algorithm also plays a key role in reducing HIV misdiagnosis rates, and a comprehensive approach to quality assurance of HIV testing is critical whether for initial diagnosis or for verification purposes [77, 78]. Implementation and operational aspects of retesting for verification may present complexities, for example, where HIV testing is conducted in community settings, or where health care facilities do not have an another person qualified to administer retesting for verification. We suggest that program managers proactively design the implementation of retesting for verification to overcome any hurdles to operationalizing the strategy. For countries adopting a phased approach, retesting for verification may be prioritized to testing sites that appear to indicate poor performance on external quality assessments and areas where higher level of discordant results are observed. We also recommend that countries review their data, consider the long term financial, personal, and societal costs associated with not retesting for verification, and include retesting for verification as an integral quality assurance component in the development or revision of their “Treat All” plans.
